# Supplementary material for: Comparison of Long-Term Complications of COVID-19 Illness among a Diverse Sample of Children by MIS-C Status
Source: Int J Environ Res Public Health. 2022 Oct 17;19(20):13382. doi: 10.3390/ijerph192013382 (PMC9603408; doi:10.3390/ijerph192013382)
Supplement: Supplementary file 1 [file ijerph-19-13382-s001.zip › ijerph-1941534-supplementary.pdf]

Supplemental Table S1. Sensitivity analysis for missing data.

	MIS-C (N=26)			Non-MISC (N=286)		
	Missing (N=4)	No missing (N=22)	P-value	Missing (N=46)	No missing (N=240)	P-value
Age at diagnosis, years, mean (SD)	6.25 (2.5)	10.69 (5.23)	0.127	6.43 (6.04)	6.55 (5.96)	0.905
Boys, n (%)	2 (50.0)	11 (50)	1.0	24 (52.17)	141 (58.75)	0.41
Race/ethnicity, n (%)			0.882			0.054
Non-Hispanic White	1 (25.0)	6 (27.27)		15 (32.61)	42 (17.5)	
Non-Hispanic Black	1 (25.0)	6 (27.27)		6 (13.04)	47 (16.4)	
Hispanic	1 (25.0)	8 (36.36)		21 (45.65)	140 (58.33)	
Other/unknown	1 (25.0)	2 (9.09)		4 (8.69)	11 (4.58)	
Education			0.399			0.177
Preschool	2 (50.0)	3 (13.64)		31 (67.39)	127 (52.92)	
Elementary school	2 (50.0)	11 (50.0)		4 (8.69)	53 (22.08)	
Middle school	0 (0)	3 (13.64)		4 (8.69)	22 (9.17)	
High school and above	0 (0)	5 (22.73)		7 (15.22)	37 (15.42)	
Insurance status			0.380			0.001
No insurance	0 (0)	1 (4.54)		2 (8.33)	5 (2.13)	
Government insurance	1 (25.0)	14 (63.64)		10 (41.67)	181 (75.42)	
Private insurance	3 (75.0)	7 (31.82)		12 (50.0)	49 (20.42)	
Hospitalization, n (%)	4 (100)	21 (95.5)	1.0	38 (82.60)	203 (84.58)	0.736
Hospital length of stay, days, mean (SD)	6.67 (5.0)	8.11 (1.20)	0.524	4.14 (0.91)	5.76 (1.14)	0.528

[a] T-test for continuous variable and chi-square analysis or Fisher's exact for categorical variable

Supplemental Table S2. Comparison of academic and physical activity at school by MIS-C status.

	**Total**	**MIS-C**	**Non-MISC**	**P-value[a]**
Returned to school or daycare[b]				0.999
Yes	147 (93.04)	21 (95.45)	126 (92.65)	
No	11 (6.96)	1 (4.55)	10 (7.35)	
School format[b]				0.040
Completely online	37 (23.13)	7 (33.33)	30 (21.58)	
Completely in-person	96 (60.0)	14 (66.67)	82 (58.99)	
Hybrid	27 (16.88)	0 (0)	27 (19.42)	
Academic performance changed since pandemic[b]				0.267
Better	18 (10.98)	0 (0)	18 (12.5)	
Worse	38 (23.17)	5 (25.0)	33 (22.92)	
No change	108 (65.85)	15 (75.0)	93 (64.58)	
Physical activity performance changed since pandemic[c]				0.158
Yes	24 (32.0)	6 (54.55)	18 (28.12)	
No	51 (68.0)	5 (45.45)	46 (71.88)	

[a] Chi-square analysis or Fisher's exact for categorical variable

[b] Asked only if a child enrolled in school or daycare

[c] Asked only if a child participated in any athletics or physical activity in or outside of school
